# Identification of on-target mutagenesis during correction of a beta-thalassemia splice mutation in iPS cells with optimised CRISPR/Cas9-double nickase reveals potential safety concerns

**DOI:** 10.1063/1.5048625

**Published:** 2018-12-03

**Authors:** Suad Alateeq, Dmitry Ovchinnikov, Timothy Tracey, Deanne Whitworth, Abdullah Al-Rubaish, Amein Al-Ali, Ernst Wolvetang

**Affiliations:** 1 Australian Institute for Bioengineering and Nanotechnology, The University of Queensland, St Lucia, QLD 4072, Australia; 2 Department of Biochemistry, College of Medicine, Imam Abdulrahman Bin Faisal University, Dammam 31451, Kingdom of Saudi Arabia; 3 School of Veterinary Science, The University of Queensland, Gatton, QLD 4343, Australia; 4 Department of Internal Medicine, College of Medicine, King Fahd Hospital of the University, Imam Abdulrahman Bin Faisal University, Dammam 31451, Kingdom of Saudi Arabia

## Abstract

Precise and accurate gene correction is crucial for enabling iPSC-based therapies, and Cas9-Nickase based approaches are increasingly considered for *in vivo* correction of diseases such as beta-thalassemia. Here, we generate footprint-free induced pluripotent stem cells from a patient with a beta-thalassemia mutation *(*IVSII-1 G > A) and employ a double Cas9nickase-mediated correction strategy combined with a *piggyBac* transposon-modified donor vector for gene correction. Our approach further aimed to minimize the formation of adjacent single-strand breaks at the targeted allele through the destruction of the binding site for one guide and the use of a synonymous protospacer adjacent motif blocking mutation (canonical PAM sequence 5'-NGG-3' is changed to 5'-NCG-3', where N indicates any nucleobase) for the other guide. We show that this strategy indeed not only permits bi-allelic seamless repair of the beta-globin gene splice site mutation and negligible off-target mutagenesis or re-editing of the targeted allele but also results in unexpected on-target mutagenesis with some guide RNAs (gRNAs) in several targeted clones. This study thus not only validates a framework for seamless gene correction with enhanced specificity and accuracy but also highlights potential safety concerns associated with Cas9-nickase based gene correction.

## INTRODUCTION

Rapid progress in the development of nuclease-based genome editing tools, particularly with respect to the CRISPR-Cas systems (Wright *et al.*, 2016), holds great potential for induced pluripotent stem cell (iSPC)-based gene therapy for monogenic diseases such as beta-thalassemia (Ou *et al.*, 2016 and Midic *et al.* 2017). Beta-thalassemia is caused by mutations in the adult beta-globin gene (*HBB*) and is one of the most prevalent monogenic blood disorders worldwide (Modell and Darlison, 2008). A number of studies have successfully used programmable engineered nucleases, such as CRISPR/Cas9 nuclease (Cas9WT), for the targeted repair of the disease-causing mutation in iPSCs derived from patients with beta-thalassemia (Xie *et al.*, 2014; Song *et al.*, 2015; Xu *et al.*, 2015; Niu *et al.*, 2016; Yang *et al.*, 2016; and Wattanapanitch *et al.*, 2018). However, unwanted on-target (Merkle *et al.*, 2015 and Kosicki *et al.*, 2018) and off-target induced mutagenesis (Bolukbasi *et al.*, 2016) is frequently associated with Cas9WT activity. To fully realize the therapeutic and disease-modelling potential of genome editing in pluripotent stem cells (PSCs), the process needs to be highly precise (i.e., limit off-target events) and accurate (i.e., limit undesired on-target re-editing events). To minimize the potential for adverse off-target mutagenic activity associated with DNA double-strand breaks (DSBs) induced by Cas9WT, a double-nicking strategy was devised (Mali *et al.*, 2013 and Ran *et al.*, 2013a). However, the targeted site remains susceptible to undesired on-target modifications following gene correction due to the iterative cycles of Cas9 activity (Merkle *et al.*, 2015 and Paquet *et al.*, 2016) related to competition and cooperation of homology-directed repair (HDR) and non-homologous end joining (NHEJ) during the repair of composite on-target DSB (Kass and Jasin, 2010 and Vriend *et al.*, 2016). To address this concern, a donor strategy design was used which prevents the formation of adjacent nicks following HDR events (Merkle *et al.*, 2015) and the use of a donor template that inserts a selection cassette at either one of the guide-binding sites or between the guide-binding sites of a pair (Merkle *et al.*, 2015). HDR accuracy can theoretically be further improved by introducing a single nucleotide polymorphisms (SNP) in the protospacer adjacent motif (PAM) sequence at the binding site for one of the guides of the pair set, as shown by Paquet *et al.* (Paquet *et al.*, 2016) for Cas9WT. The effectiveness of both strategies relies on the high-fidelity repair of the single-strand breaks (SSBs). However, the mutagenic activity of monomeric nickases varies between cell types, gene locus, and the strategy used (Merkle *et al.*, 2015 and Miyaoka *et al.*, 2016), and data on the accuracy and efficacy of these strategies for gene correction in human iPSCs remain limited. Here, we combine all of the above approaches to correct a *HBB* gene splice mutation in beta-thalassemia iPSCs, a locus that appears particularly susceptible to off-target activity for some sgRNA sequences (Cradick *et al.*, 2013 and Xu *et al.*, 2015).

We demonstrate that this combined strategy can not only indeed prevent undesired on-target indels for some guides but also reveal an unexpectedly high rate of mutagenesis associated with the stand-alone action of Cas9n at one of the sgRNA sites, as indicated by the presence of an indel at the binding site of one of the guides. While our data thus show that biallelic seamless gene correction of a beta-thalassemia-causing splice site mutation is possible, the vast majority of mono-allelically targeted clones possess an indel at the guide binding site of the untargeted allele, indicating a high level of either Cas9DN- or Cas9n-mediated activity.

Collectively, our approach not only validates the utility of a combined Cas9DN-mediated strategy for achieving biallelic seamless gene correction with enhanced specificity but also identifies unexpected mutagenic effects of Cas9-nickase associated with some guides using this strategy. This highlights that despite inbuilt safety features, CRISPR Cas9-approaches need to be carefully evaluated for each gene and guide before progressing to *in vivo* gene correction in patients.

## RESULTS

### iPSC generation, gene correction strategy, and guide-pair selection

**FIG. 1.**
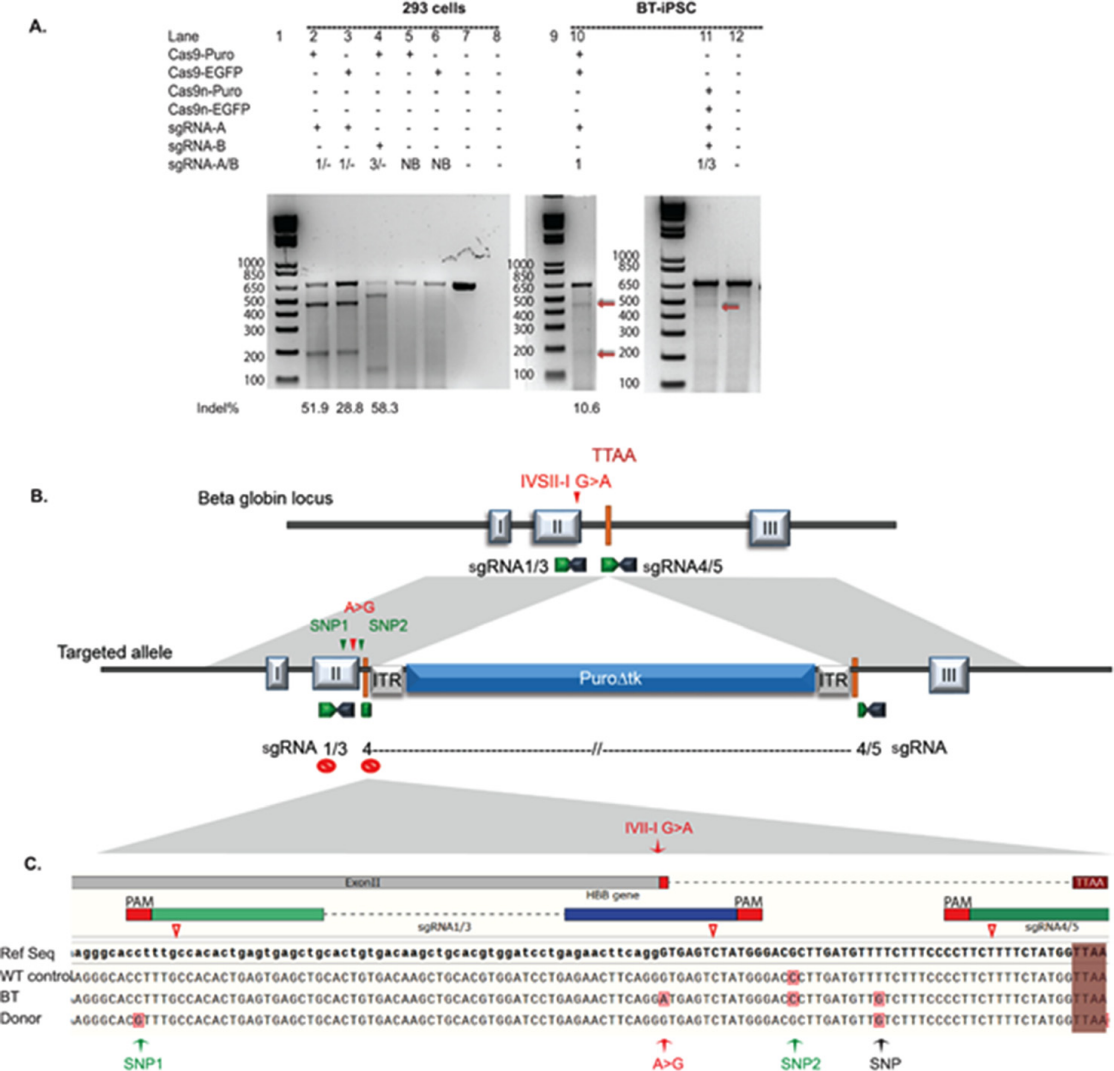
Guide validation and the gene editing strategies used in the genetic correction of the splice junction mutation in BT-iPSCs. (a) Observed guided Cas9 activity as demonstrated by the Surveyor assay: (1) of HEK293FT cells transfected with either sgRNA-Cas9WT-EGFP- or sgRNA-Cas9WT-puromycin-expressing vectors (lanes 2–4) and sgRNA-Cas9WT expressing non-binding guide sequence (Lanes 5 and 6); (2) upon transfecting BT-iPSCs with sgRNA1-Cas9WT-EGFP (lane 10) or with the dual nickase system (lane 11). A SV1 primer was used to generate the PCR fragment spanning the targeted site. Lanes 7 and 12: undigested PCR product; Cas9 cleavage site (red Δ). (b) Schematic representing gene-editing strategy design using two different guide pairs. The PAM sequence was inactivated at the targeted allele in the first pair (sgRNA 1/3), while the binding site for the first guide (sgRNA4) in the second pair (sgRNA4/5) was destroyed following the HDR event by the placement of the selection cassette. (c) Schematic of the location of the SNPs introduced in the donor template which include (1) a PAM blocking mutation to prevent CRISPR-Cas9n from generating a closely spaced single strand breaks (SSBs) in the donor template or the corrected allele when sgRNA1/3 is used (SNP1, green arrow); (2) a tracer SNP to validate that the modification occurred through recombination (SNP2, green arrow); and (3) a base substitution that corrects the disease-causing mutation (red arrow). The black arrow represents a SNP variant in BT-iPSC. (See Fig. S1 for more details.) EGFP, enhanced green fluorescent protein; Cas9n, Cas9nickase; Puro, puromycin; sgRNA-A/B: sgRNA-A and sgRNA-B; and NB, non-binding guide sequence.

Footprint-free iPSCs were generated from fibroblasts of an individual homozygous for the beta-thalassemia mutation (IVSII-1 G > A) and a control, as described by Yu *et al.* (2009). The pluripotency of the derived clones was confirmed by their ability to form tissues from all three embryonic germ layers in teratoma assays and the expression of pluripotency markers (not shown). To seamlessly correct the disease-causing IVII-I G > A splice mutation, we wished to insert a dual selection *piggyBac* transposon-based excisable cassette (Yusa, 2013) into two different loci located in the vicinity of the disease-causing mutation [Fig. 1(b)] in intron II of the *HBB* gene in beta-thalassemia iPSCs [Fig. S1(a)] using a double Cas9n approach. To minimize DNA modifications brought about by the iterative cycle of Cas9 activity following HDR events, we combined two previously validated safety features aimed at preventing the formation of closely spaced SSBs at the targeted site. First, the placement of the selection cassette (Merkle *et al.*, 2015) in the targeting construct was designed to destroy the binding site for one guide, and a synonymous PAM blocking mutation (NGG > NCG) was used for one guide of the other pair [sgRNA1; Figs. 1(b) and 1(c)]. A “tracer” SNP to detect targeted clones and a base change to correct the disease-causing mutation were also introduced in the left homology arm of the donor template, as shown in Fig. 1(c). The PAM sequence was inactivated at the targeted allele in the first pair (sgRNA 1/3), while the binding site for the first guide (sgRNA4) in the second pair (sgRNA4/5) was destroyed following the HDR event by the placement of the selection cassette. Multiple pairs of sgRNAs were screened for their ability to efficiently introduce DNA breaks at different distances close to the *HBB* mutation site or upstream of the *piggyBac* TTAA insertion site [Fig. S1(a)]. The offset distance for different pairs varied between −7 and +51 bp [Fig. S1(a)]. All five pairs were assessed for their ability to induce indels at the targeted site in HEK293 cells. To facilitate monitoring of the transfection efficiency via EGFP fluorescence and drug selection, two different expression vectors were used to clone guides for each pair: PX461 (EGFP) and PX462 (puromycin). Twenty-four hours after selection, the majority of puromycin-resistant cells expressed green fluorescent protein (GFP) [Fig. S1(b)]. A relatively high occurrence of indels was observed for all pairs, as assessed by Surveyor assay [Fig. S1(c)]. We selected guide pairs sgRNA1/3 and sgRNA4/5 for subsequent experiments as they had the highest quality score as indicated by the sequence analysis using the CRISPR Design tool (http://crispr.mit.edu/). Following nucleofection of these guides into beta-thalassemia iPSCs (BT-iPSCs), the surveyor nuclease assay revealed that both Cas9DN (sgRNA1/3-Cas9n) and sgRNA1-Cas9WT induced indels at the targeted locus [Fig. 1(a)] but that, as expected, the full nuclease was more efficient.

### Gene targeting of the disease-causing mutation in iPSCs

Multiple independent nucleofection experiments were performed using both strategies, as illustrated in Table S1. The observed absolute targeting efficiencies of 7.5 × 10^−6^ and 1.95 × 10^−5^ for sgRNA1/3 and sgRNA4/5 (Tables S1A6 and A5) were comparable to the previously reported double nickase mediated targeting efficiencies across 13 gene loci (6 × 10^−6^) (Merkle *et al.*, 2015). To evaluate the efficiency of our strategies for mediating HDR events, a total of 50 puromycin-resistant clones were selected from three different transfection experiments (Table S1) and screened using the polymerase chain reaction (PCR)--based strategy described by Yusa (2013) [Fig. S2(a)]. There was no amplification of the targeted allele in clones generated with the linear donor vector, indicating that their drug resistance was due to the random integration of the selection cassette [Figs. S2(b) and S2(c)]. Half (52%) of the clones obtained using a circular donor vector had one targeted allele, 16.67% showed a possible targeting event at both alleles, and the remainder 31.26% showed random integration of the donor vector and were excluded from further analysis (Table I). The probability of obtaining a targeting event at one or two alleles was clearly dependent on the guide pairs used (p = 0.011, Fisher's exact test). These data indicate that the efficiency of HDR is influenced by the efficiency of the guide pair, the location of the nick sites relative to the insertion site and to each other, or by other sequences and locus-dependent factors. At this stage, we had already noted the considerable variation in the band size of amplicons obtained from the original allele [Fig. S2(b) and S2(c)], suggesting the presence of a spectrum of on-target indels induced by Cas9DN at this allele. Overall, 81.25% of the resistant clones possessed at least one targeted allele, indicating that our strategy permitted efficient *HBB* gene targeting in iPSCs. Screening for random integration was carried out using a PCR-based strategy [Fig. S2(d)] as suggested by Yusa (2013). A total of 33/48 clones were identified that were devoid of random integrations. The screen revealed a higher number of random integrations associated with sgRNA1/3 (42.8%) compared to sgRNA4/5 (20.59%) (Table I). The sequence analysis of the left arm in selected clones indicated the correction of the disease-causing mutation and the introduction of the two intentionally introduced SNPs, confirming that gene modification had occurred through HDR [Fig. S2(f)]. Interestingly, a high frequency of PAM base substitution was incorporated at the targeted allele, in 11/11 clones (6 biallelic and 5 monoallelic) transfected with sgRNA4/5 despite the fact that it is located 100 bp upstream of the sgRNA4 cut site, a supposedly suboptimal distance for allelic conversion (Elliott *et al.*, 1998). This finding is consistent with a longer gene conversion tract observed with the dsDNA donor template (Elliott *et al.*, 1998) albeit at a higher frequency of concurrent base substitution occurring at 24, 39, and 100 bp upstream from the DNA break site (sgRNA4). Importantly, no on-target modification was detected at the DNA binding site for either sgRNA1 or sgRNA3. Surprisingly, sequencing of the right arm revealed the presence of deletions at the binding site for one of the guides (sgRNA5) in one clone [Fig. S2(g)]. The presence of DNA modification at a single guide binding site (when another sgRNA seed site is destroyed by insertion of the selection cassette) suggests that, contrary to what is widely believed, DNA nicks can be repaired through mutagenic pathways in iPSCs.

**TABLE I.** Frequency of clones with targeted alleles in puromycin-resistant clones. R, random integration; M, different band size (compared to control), indicating either deletion or insertion or targeting at only one arm; T/R, mono or biallelic targeted event with random integration; T, targeted events. (See Fig. S2 for more details on the screening strategy and Table S1 for the experimental design.) Number of clones screened are represented in bold.

	Clones excluded			Clones included			% of resistant clones with a targeted event
Guide pair	(R/M)	Monoallelic/R	Biallelic/R	Monoallelic	Biallelic	Number of clones screened	T/R or T
Circular donor							
sgRNA1/3	**6 (5/1)**	**1**	…	**6**	**1**	**14**	
%	42.86	7.14	…	42.86	7.14		57.14
sgRNA4/5	**3 (2/1)**	**2**	**3**	**19**	**7**	**34**	
%	8.82	5.88	8.82	55.88	20.59		91.17
Total	9	3	3	25	8	48	
%	18.75	6.25	6.25	52.08	16.67		81.25
Linear donor							
sgRNA1/3	**2**	…	…	…	…	**2**	
						**50**	

### Assessment of undesired Cas9-mediated on- and off-target activity

All the monoallelically targeted clones (n = 25) were next screened for the presence of an indel at the untargeted allele and for alterations in the left and right arm DNA regions of the guide binding sites at the targeted allele. To this end, we employed the heteroduplex mobility assay (HMA), an efficient high-throughput method to screen for Cas9-induced allelic alterations (Zhu *et al.*, 2014). Polyacrylamide gel electrophoresis (PAGE)-based genotyping relies on the detection of a heteroduplex formed between wild-type and mutant DNA fragments resulting from Cas9-mediated indels at the screened locus. Multiple heteroduplex bands were observed for some clones (3/25) [Fig. S3(c)], indicating that such clones were genetically heterogeneous. This heterogeneity may have resulted from mosaicism due to a non-unicellular source or may indicate modification in a subset of cells as a result of continuous nuclease activity, as suggested in a previous study (Merkle *et al.*, 2015). However, the majority of the clones displayed a pattern consistent with the presence of only one allele, thus confirming clonality and limited Cas9 re-editing events in the majority of clones.

The data also indicate that indels were present at the untargeted allele in all 25 monoallelically targeted clones included in the screen [Fig. S3(c)]. This high rate of indels at the untargeted allele was observed for both guide pairs used to target the locus and consisted of different types of mutations, as revealed by the unique band patterns observed for each clone [Fig. S3(c)] and sequence analysis [Figs. S3(d) and S3(e)]. For guide pair sgRNA4/5 with an offset of 5 bp, we observed a mutation spectrum that consisted of a range of deletions that varied between 5 and 54 bp and that mostly affected the intervening sequence between the guide binding sites [Fig. S3(d)]. We noticed the presence of short microhomology regions (2–4 bp) flanking the deleted sequence [Fig. S3(e)] in 67% of the clones. Usually, this pattern of deletion is associated with the c-NHEJ DNA repair pathway (McVey and Lee, 2008). Intriguingly, an inversion with deletion and insertion of 17 bp was detected in two independent clones targeted with sgRNA1/3 with an offset of 28 bp [Fig. S3(f)]. Recently, Kosicki *et al.* (2018) brought to light the complexity of on-target collateral DNA damage resulting from the use of Cas9WT. The reported DNA damage included large deletions, inversion, insertion, and translocation. Collectively, these data may suggest that the length of the overhang, as well as locus or sequence-dependent characteristics, is likely to play a role in determining the mechanism of repair.

We considered that the unexpectedly high rate of on-target mutagenesis observed at the untargeted allele could have been due to the amount of Cas9 plasmid used for transfections, as previous evidence suggested that increasing the plasmid dose can increase the frequency of on-target mutations (Fu *et al.*, 2013 and Merkle *et al.*, 2015). Although we cannot exclude this possibility, we did not decrease the amount of the Cas9 expressing vector, given that the indel occurrence in the bulk iPSC population was below the detection limit of the Surveyor nuclease assay [Fig. 1(a)]. The strategy of antibiotic selection and clonal expansion can capture rare events that are possibly below the detection limit of methods commonly used to detect mutations in a heterogeneous cell population. Furthermore, the data indicate that the expression of vector-borne genes is quite low, as evidenced by the weak fluorescent signal generated by the expression of the EGFP-expressing Cas9 vector.

**FIG. 2.**
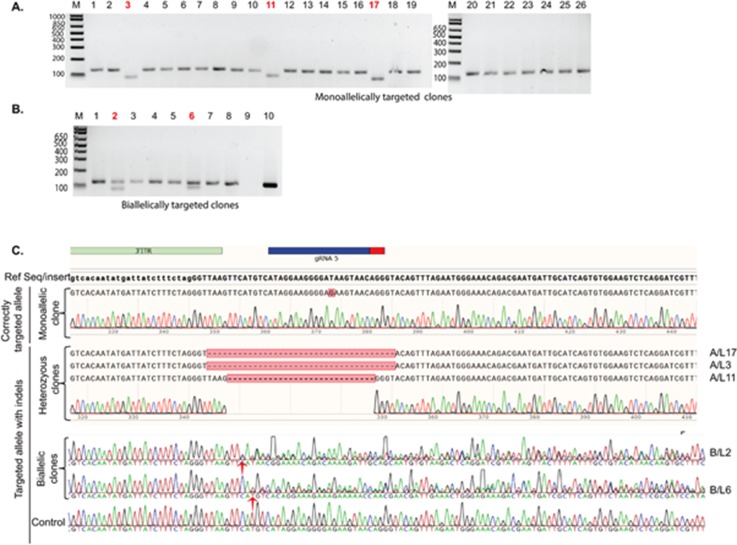
Unintended nickase-induced DNA modifications identified at the sgRNA5 binding site in gDNA extracted from targeted clones. [(a) and (b)] The observed band pattern for PCR amplicons spanning the guide binding site for (a) 25 clones with a monoallelically targeted allele (Mo-T) (lane A-26: positive control using a correctly targeted clone confirmed by sequencing) and (b) 8 clones with biallelic targeting (Bi-T) [lane B-9: negative control (BT-iPSCs) and lane B-10: donor vector]. Band patterns indicate deletions in three Mo-T and two in Bi-T clones (highlighted in red). (c) Sequence analysis of the sgRNA5 binding locus in selected clones [red colored number in panel (a)] confirms the presence of deletions at the guide binding site at the targeted allele. The chromatograms become noisy with mixed sequencing traces observed downstream of the guide binding site (red arrow) in two homozygous clones, confirming the presence of mutation regions at the site. (See Fig. S3 for the HMA screening strategy of undesired on- and off-target mutagenesis.)

No mutation was detected as a result of nickase-induced SSBs at either the sgRNA3 or the sgRNA4 binding sites in the targeted alleles. Remarkably, on-target allelic modification was identified at the sgRNA5 binding site in the right arms of both heterozygous (3/19) and homozygous (2/7) clones [Figs. 2(a) and 2(b)], despite the separation of the guide pair by the insertion of the selection cassette. These observations suggest that in contrast to the findings of Merkle *et al.* (2015), on-target mutagenesis does not become negligible for one guide (in this case with sgRNA5) by the separation of the SSB sites and indicates single nickase mutagenic activity. The presence of indels was further confirmed using Sanger sequencing [Fig. 2(c)].

To examine potential off-target mutagenesis induced by Cas9n, the five top-scored genomic sites that bear high sequence homology to the sgRNA-targeted region were screened for the presence of mutations. Ten potential DNA sites for each guide pair (five sites per sgRNA) were screened in three different clones, one generated using the sgRNA1/3 and two using the sgRNA4/5 guide pairs. The presence of heteroduplex bands was used to assess the presence of allelic mismatches (relative to the iPSC parental line) at the screened DNA site. No off-target activity at the selected loci was detected as shown by the presence of only a single band, indicating that no indels were present and only homoduplexes were formed between amplicons [Fig. S3(g)]. The presence of heteroduplexes and the absence of a homoduplex at one site (G4/P2) for sgRNA4 in two screened clones are not due to off-target activity [Fig. S3(g)]. This pattern rather suggests the retention of heterozygosity at this locus, as a similar pattern was observed in the parental clone. The sequence was further verified by Sanger sequencing for three randomly selected off-target sites (P1–5) for each gRNA, including the DNA site for off-target P2/sgRNA4. Sequence analysis indeed revealed heterozygosity resulting from a three base deletion variant at one allele. This finding highlights the importance of including the parental line as a control to detect heterozygosity at the analysed DNA sites in PAGE-based genotyping.

Collectively, we identified six correctly bi-allelically targeted clones from eight homozygous clones (between sgRNA1/3 and sgRNA4/5-assisted corrections) but found that all the heterozygous clones carried an indel at the untargeted allele. Our data further show that, although the strategy was effective in mitigating indels for some guide sequences, the effectiveness can depend on the potential mutagenic activity of monomeric Cas9-nickase. Furthermore, the study validates HMA as an efficient approach that allows rapid screening of Cas9-induced allelic alterations.

### Seamless excision of the selection cassette in correctly targeted clones

**FIG. 3.**
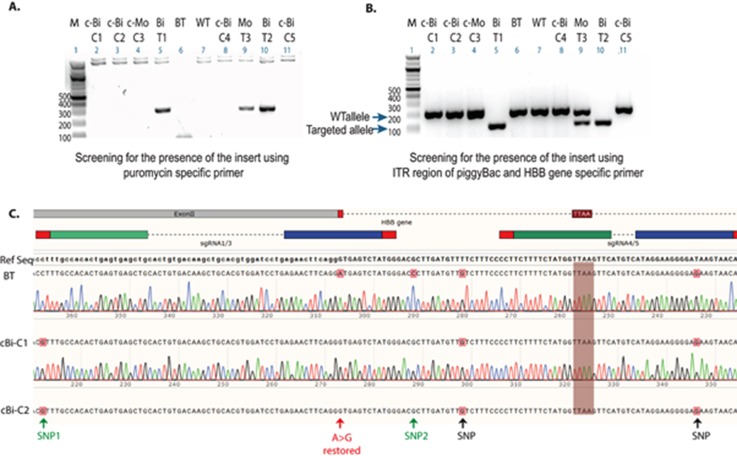
Restoration of the TTAA site following excision of the selection cassette and the absence of reinsertion. (a) Analysis of amplicons obtained by PCR for the puromycin resistance gene indicates the absence of reinsertion of the selection cassette in biallelic (c-Bi-C1 and C2) and monoallelic (c-Mo-C3) gene-corrected clones following transfection with transposase and single cell cloning by limiting dilution (from original targeted iPS clones Bi-T1, Bi-T2, and Mo-T3). (b) Analysis of amplicons obtained by multiplex PCR [PCR strategy outlined in Fig. S4(a)] from selected clones indicates selection cassette excision in clones C1, C2, and C3 derived from clones T1, T2, and T3. (c) Sanger sequencing of the corrected allele confirms the restoration of the TTAA site and the presence of all three SNPs at the expected designated locations. BT, beta-thalassemia iPSCs; Bi-T, biallelically targeted clones; Mo-T, monoallelically targeted clones; c-Bi-C, corrected biallelically targeted clones with the restored TTAA site; and WT, wild-type iPSCs control. (See Fig. S4 for more details.)

The main rationale for using the *piggyBac* transposon system for gene correction was that it should allow for a seamless excision of the selection cassette upon transfection with transposase (Yusa, 2013). In our hands, negative selection with Fialuridine (FIAU) exhibited limited efficiency, perhaps because the selection cassette is inserted into a locus (*HBB*) that is not expressed in iPSCs, or indicating that the cassette (or its negative selection component) was silenced. A similar observation was reported in a study where a hygromycin selection cassette driven by the PGK promoter was inserted into the first intron of the *HBB* gene in iPSCs (Zou *et al.*, 2011). To overcome this, an alternative strategy for enriching and isolating iPSCs with excision events was adopted [Fig. S4(b)] based on an approach reported by McCormick (1987) and Miyaoka *et al.* (2014). Cells from two different bi-allelically targeted clones (BiT-C1/sgRNA1/3 and BiT-C2/sgRNA4/5) and one mono-allelically targeted clone (MoT-C3/sgRNA1/3) were transfected with a transposase expression plasmid. Enrichment for excision events followed by limited dilution single cell cloning [Figs. S4(c)–S4(e)], next enabled the isolation of iPSC clones with biallelic excision, as indicated by the presence of a single band of the expected size [Fig. 3(b)]. The sequence of the amplicon spanning the selection cassette insertion site from the gene-corrected clones confirmed the precise biallelic restoration of the TTAA site after excision [Fig. 3(c)], and the absence of cassette reinsertion was confirmed by PCR for the puromycin resistance gene [Fig. 3(a)].

Two clones that were seamlessly corrected at both alleles and one heterozygous clone with one allele carrying a 20 bp deletion (sequence shown in Fig. S3F, Mo-T3) were next subjected to karyotype analysis. Each of these clones displayed a normal karyotype (data not shown) and expressed OCT4 and TRA-1-60 pluripotency markers. We conclude that seamless correction of both mono- and bi-allelically targeted iPSCs is achievable with this strategy. The three corrected clones were next subjected to further experimentation and assessment of the corrective strategy.

### CRISPRa mediated activation of the HBB gene

**FIG. 4.**
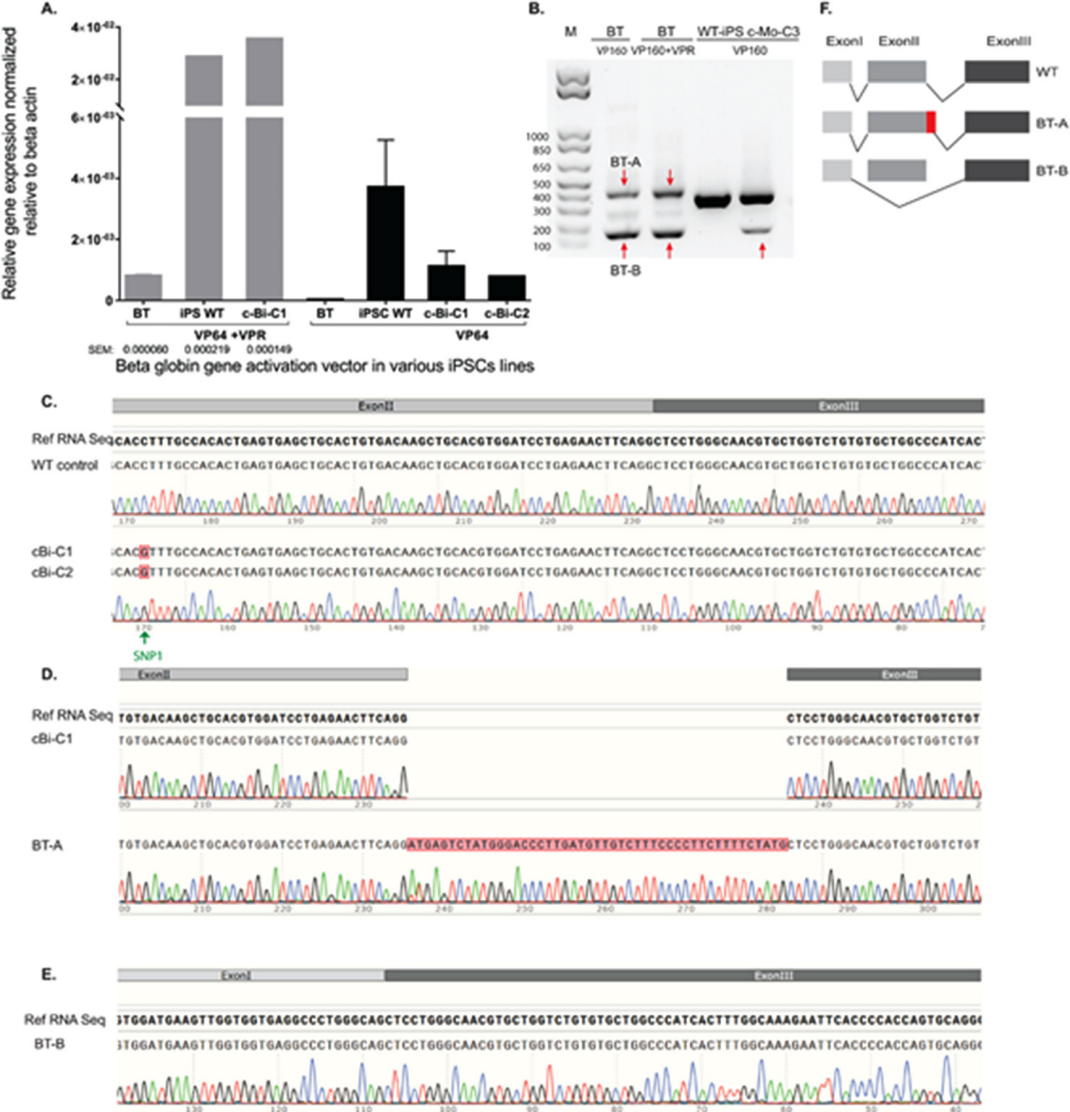
Gene expression and sequence analysis of RNA products derived from the activated HBB gene in iPSCs using a dCas9-activator. (a) The levels of *HBB* gene expression in iPSCs determined 72 h after transfection with a Cas9-mediated activator and *HBB* sgRNAs in WT, BT, and corrected beta-thalassemia iPSCs (two independent replicates ± SEM). (b) Analysis of PCR amplicons generated from cDNA from the indicated iPSCs using primers spanning intron I and II of the *HBB* gene. Bands that differ in size from the expected 397bp (red arrows) indicate aberrant splicing products. (c) Sequence analysis of PCR products confirms the restoration of normal splicing following the seamless gene correction of the mutation. The presence of the PAM blocking mutation (SNP1) provides further evidence that the product was derived from the corrected allele. [(d) and (e)] Sequence analysis of the PCR products obtained from BT-iPSCs and Mo-C3 [indicated with red arrows in (b)] reveals that mutation at the IVSII splice site results in the formation of aberrantly spliced products that include (d) the use of an alternate splice site located 47 bp downstream of the mutation or (e) exon II skipping. (f) Schematic of the splicing process identified in wild-type and mutant *HBB* genes.

Because achieving efficient *HBB* gene expression in erythroid cells derived from gene-edited iPSCs *in vitro* can be difficult (Zou *et al.*, 2011; Ma *et al.*, 2013; and Xie *et al.*, 2014), costly, and time consuming, we decided to use a CRISPRa (CRISPR activation) approach (Dominguez *et al.*, 2016) to assess the restoration of normal RNA splicing in corrected iPSCs. The induction of RNA expression of the beta-globin gene was first explored in HEK293FT cells. Eight sgRNAs that span the proximal promoter region of the *HBB* gene were designed [Fig. S5(a)]. Combining multiple guides (sgRNA_a-b_) with dCas9VP64 or dCas9VP160 systems [VP16_(n)_] efficiently induced *HBB* RNA expression [Figs. S5(b) and S5(c)]. However, increasing the number of repeats of the VP16 activation domain from four to ten did not substantially increase RNA expression levels of the *HBB* gene [Fig. S5(c)], a finding consistent with a previous study (Chavez *et al.*, 2016). A significantly higher level of gene induction was achieved by adding a vector expressing a hybrid activation domain (dCas9-VPR: VP64, p65, and Rta) (Chavez *et al.*, 2015) to the transfection mix [sgRNA_a-b_-dCas9-VP16_(n)_] using a lower dosage of vectors expressing the guide combinations [Fig. S5(c)]. Based on quantitative polymerase chain reaction (q-PCR) data for HEK293 cells, we next delivered selected active guide combinations to WT-iPSCs in the presence and absence of the dCas9-VPR expressing vector. As expected, a significantly higher level of *HBB* gene activation was achieved in the presence of the VPR expressing vector in transfected iPSCs [Fig. 4(a)]. Overall, these results indicate that it is possible to induce *HBB* expression using VP16_(n)_ systems but that increasing the VP16 tandem repeat number in the activation domain does not lead to a significant difference in the induction of *HBB* expression for the guide combinations assessed under these experimental conditions. Our data also support the enhanced activation potency of the hybrid VPR system (Chavez *et al.*, 2015 and 2016).

This CRISPR activation approach in iPSCs with the beta-thalassemia splice junction mutation permitted detection of the abnormally spliced transcript, albeit at a lower level than activated *HBB* in wild-type control iPSCs [Figs. 4(a) and S5(d)]. DeadCas9-VP64-induced HBB expression levels varied between 36 and 237-fold depending on the presence or absence of dCas9-VPR, respectively. To further investigate the nature of this spliced transcript, a primer pair that spans introns I and II was used to PCR amplify the region from cDNA samples. Two bands were observed for beta-thalassemia cells and for monoallelically targeted clones with the untargeted allele carrying a 20 bp deletion [Fig. 4(b)]. This indicates that abnormal splicing events occurred in the presence of either a point mutation or deletion affecting the splice donor site which include GU sequence at the 5' end of the intron or the adjoining region. Sanger sequencing of the PCR products revealed the occurrence of a splicing event 47 bp downstream of the original splice site [Fig. 4(d)] and splicing with exon II skipping [Fig. 4(e)], which is in agreement with the study by Treisman *et al.* (1982) in HeLa cells. Overall, these data confirm that it is possible to model the splice junction defect in iPSCs with a beta-thalassemia mutation using the CRISPRa system.

### Beta-globin gene expression in iPSCs after gene correction

To assess the restoration of the normal splicing pattern after gene correction and excision of the selection cassette, gene-edited iPSCs were transfected with the sgRNA_(3–8)_-dCas9-VP64/VPR vector combination, and the gene expression levels were assessed using a primer pair that spans the intron II region of the *HBB* gene. The data consistently demonstrated the restoration of normal splicing [Figs. 4(a) and S5(d)]. These observations indicate that gene-corrected beta-thalassemia iPSCs restored expression of *HBB* mRNA and that the presence of the introduced SNP near the splicing regions did not affect the splicing process. To further confirm and assess the type of the splice products of the *HBB* gene, PCR amplicons were generated from cDNA obtained from transfected gene-edited iPSCs. Similar band sizes were observed upon comparing the products obtained from transfected corrected iPSCs to those of the transfected wild-type control [Fig. S5(e)]. Sequencing of PCR amplicons provided confirmation of the restoration of normal splicing in gene-corrected iPSC lines, including the detection of the introduced synonymous SNP in the transcript [Fig. 4(c)]. Notably, the CRISPRa approach in iPSC, HEK293, and K562 cells resulted in the formation of beta-globin transcripts with similar sequences and splicing patterns to that observed in untransfected K562 cells [Figs. S5(f) and S5(g)]. We also confirmed that *HBB* gene activation in K562 cells resulted in an increase in gene [Fig. S5(h)] and protein expression [Fig. S5(i)]. Collectively, these data suggest that CRISPRa/gRNA delivery recapitulates the natural transcription, splicing patterns, and gene correction restored normal splicing and that no cryptic splice site was created by the introduced SNPs. Therefore, we conclude that the CRISPR/Cas9 activation-based strategy is useful for modeling the splicing defect and is effective in assessing the restoration of normal RNA processing in gene-corrected beta-thalassemia iPSCs.

## DISCUSSION

Precise genome-engineering is needed for accurate iPSC-based modelling of genetic disease, for the provision of safe gene-edited cells in regenerative medicine (Musunuru, 2013 and Maeder and Gersbach, 2016), and for future *in vivo* gene correction. Multiple studies have reported successful gene correction of beta-thalassemia mutations using Cas9WT in iPSCs (Xie *et al.*, 2014; Song *et al.*, 2015; Xu *et al.*, 2015; Niu *et al.*, 2016; and Yang *et al.*, 2016), but Cas9WT is commonly associated with on-target (Merkle *et al.*, 2015 and Kosicki *et al.*, 2018) and off-target mutagenesis (Bolukbasi *et al.*, 2016). Cooperative strategies [i.e., double nickase (Cas9n) such as is used in the DN strategy], which induce DSBs at the on-target site but only single nicks at single gRNA off-target sites, should be advantageous for precise DNA modification (Mali *et al.*, 2013 and Ran *et al.*, 2013a). Preventing the iterative cycles of dual Cas9n activity by destroying the binding site for one of the guide pairs that target the locus of interest (Merkle *et al.*, 2015), the introduction of a blocking SNP in the PAM sequence (Paquet *et al.*, 2016), or the use of offset DNA nicks should have theoretically limited unwanted on-target mutagenesis. Here, we show that combining these approaches with a *piggyBac* transposon excision strategy in human iPSCs indeed permits seamless gene correction of a *HBB* splice junction mutation that causes beta-thalassemia. Notably, homozygosity of the introduced PAM blocking SNP, located 100 bp upstream from the Cas9 cleavage site, was observed in all our biallelic targeted clone (sgRNA4/5), suggesting a longer conversion tract (Elliott *et al.*, 1998) under our experimental conditions. Moreover, successful *piggyBac*-mediated biallelic excision of the selection cassette was attained (Yusa *et al.*, 2011a), with full and traceless *piggyBac* transposon removal from the corrected *HBB* allele. Using this approach, we obtained an even higher percentage of targeted events as compared to a similar study that used Cas9WT in conjunction with a *piggyBac*-based approach to correct a beta-thalassemia mutation (Xie *et al.*, 2014). Despite this efficient and accurate biallelic gene correction in iPSCs, separating the guide binding sites at the targeted allele did not eliminate unwanted on-target indel frequency for one guide, indicating that SSBs induced by Cas9n can be mutagenic in human iPSCs. Moreover, the untargeted allele remained subject to unwanted Cas9-mutagenic activity in monoallelically targeted clones. Although the number of analysed clones and targeted loci was limited, we noted that the pattern of mutations obtained with different guide pairs varied. This is in agreement with earlier observations, indicating that varying offset lengths of staggered DSBs (Kim *et al.*, 2013) or protospacer sequences (van Overbeek *et al.*, 2016), can result in different mutational patterns that may be related to the different mechanisms of DNA repair evoked. Similar differences in mutation patterns brought about by varying spacer sizes were previously observed with zinc-finger nucleases (ZFNs) and transcription activator-like effector nucleases (TALENs) (Kim *et al.*, 2013). Alternatively, while not 100% safe, our strategy is clearly effective at limiting on-target mutagenesis, as indicated by the large difference in the indel frequency between the targeted and untargeted alleles, and the lack of indels at the binding site of one active guides (sgRNA3) following HDR events, in agreement with a previous study (Merkle *et al.*, 2015). Our data are also consistent with the notion that monomeric nicks are less prone to induction of indels compared to strategies that introduce DSBs (Metzger *et al.*, 2011; Ramirez *et al.*, 2012; Davis and Maizels, 2014; and Merkle *et al.*, 2015). This is further corroborated by the observed lack of off-target mutations, in agreement with other studies (Ran *et al.*, 2013a; Cho *et al.*, 2014; Shen *et al.*, 2014; and Frock *et al.*, 2015). Further improvements in safety and accuracy of HDR may be able to be achieved through the use of Cas9 variants with limited expression during the cell cycle (Howden *et al.*, 2016), the introduction of a blocking mutation in single-stranded oligo DNA nucleotide (ssODN) donors (Paquet *et al.*, 2016), the use of Cas9-based adenine base editors (Gaudelli *et al.*, 2017), or the deployment of engineered high-fidelity Cas9 variants, i.e., eSpCas9 (Slaymaker *et al.*, 2016), or SpCas9-HF1 (Kleinstiver *et al.*, 2016).

Successful restoration of a wild-type allele in gene-corrected iPSCs is usually demonstrated via differentiation of iPSCs into the cell types that express the edited gene (Merkert and Martin, 2016). To circumvent the necessity for such, often costly and time-consuming, differentiation experiments, we explored the utility of delivering a CRISPR-mediated transcriptional activator (dCas9-VP64) (Dominguez *et al.*, 2016) in undifferentiated iPSCs. We show that in beta-thalassemia iPSCs, this CRISPR activation approach permits accurate modeling of a beta-thalassemia splice junction mutation in *HBB,* similar to what was previously shown in HeLa cells overexpressing the beta-globin gene with the IVSII-1 G > A mutation (Treisman *et al.*, 1982), and facilitates ready identification of restored RNA processing in corrected cells.

In conclusion, our study has validated a combined approach for traceless gene correction with enhanced specificity that exhibits sufficient efficacy to permit simultaneous repair of both alleles in iPSCs. Collectively our data indicate that targeting-strategies aimed at eliminating unwanted indels by preventing the formation of closely spaced SSBs at the targeted allele are effective but are dependent on the mutagenic activity of each monomeric sgRNA-Cas9n, which is in agreement with a previous study in human U2OS and HEK293 cells (Tsai *et al.*, 2014). Collectively, our data thus caution that even a safety optimised dual nickase strategy can still carry a risk of mutagenic activity associated with monomeric nickase that is guide- or locus-dependent, albeit at a significantly lower level as compared to the WT Cas9 nuclease (Ran *et al.*, 2013a).

## METHODS

### Derivation of iPSCs

Dermal skin punch biopsies (3 mm^2^) were obtained from one patient homozygous for a splice junction mutation (IVSII-1 G > A) that abolishes beta-globin chain synthesis (beta^0^-thalassemia) and from one wild-type control. All primary samples were collected from donors by a trained physician after obtaining informed consent. Reprogramming of fibroblasts into foot-print free iPSCs was carried out as described by Yu *et al.* (2009) using a combination of episomal vectors that expressed *OCT4, SOX2, NANOG, LIN28, c-MYC, KLF4,* and *SV40LT* (Combination 4; plasmid# 20924: pEP4 E02S CK2M EN2L and plasmid# 20927: pEP4 E02S ET2K, a gift from James Thomson) (Yu *et al.*, 2009). All work was carried out under the approval of the University of Queensland Human Research Ethics Committee (#2012000558).

### Maintenance of cell cultures

Human embryonic kidney (HEK) 293FT cells (Life Technologies) were maintained in DMEM (Dulbecco's Modified Eagle's medium) medium supplemented with 10% FBS (fetal bovine serum), 0.1 mM Minimum Essential Medium non-essential amino acids (MEM NEAA), and 1 mM sodium pyruvate. Cells were passaged every two days using TrypLE.

K562 cells were maintained in RPMI-1640 medium supplemented with 15% FBS at a seeding density of 2 × 10^−5^ and passaged every two days.

Pluripotent stem cells were either maintained on mouse embryonic fibroblast (MEF) feeder cells in KSR-hESC culture medium (DMEM Ham's F-12 medium, 20% Knockout Serum Replacement (KSR), 1% non-essential amino acids, 2 mM L-glutamine, 0.1 mM beta-mercaptoethanol, and 100 ng/ml basic fibroblast growth factor (bFGF), all from Gibco, except for bFGF, which was from Invitrogen) or on extracellular matrix (ECM) Gel (from Sigma) in MEF-conditioned KSR-hESC medium, at 37 °C with 5% CO_2_. Subculture was performed on day 7 using manual passaging of clones. Cells were regularly tested for mycoplasma.

### CRISPR plasmids

For inducing DNA break(s), all-in-one CRISPR/Cas9 vectors system: pSpCas9(BB)-2A-GFP (PX458) (Addgene plasmid # 48138), pSpCas9(BB)-2A-Puro (PX459) (Addgene plasmid # 48139), pSpCas9n(BB)-2A-GFP (PX461) (Addgene plasmid # 48140), and pSpCas9n(BB)-2A-Puro (PX462) (Addgene plasmid # 48141) (gift from Feng Zhang), expressing both a chimeric single gRNA driven by the U6 promoter and a human codon-optimised Cas9 driven by the CBh promoter, were used (Ran *et al.*, 2013b). Guide RNA sequences with a sequence pattern GN_19_NGG encoding the targeted sequences (Table S2) were designed using the online CRISPR design tool (http://tools.genome-engineering.org) (Hsu *et al.*, 2013). The vectors were constructed as outlined in the study by Ran *et al.* (2013b). The presence of the insert was detected by PCR screening using the U6 forward primer and the complementary strand of sgRNA as a reverse primer. The correct insert sequence was further verified by Sanger sequencing.

To test the functionality of single or paired guide RNAs, cells were transfected (transfection conditions are described below) with the specified vector or combination of vectors, collected by trypsinisation after 72 h, and the Surveyor assay was carried as described by Ran *et al.* (2013b). Briefly, DNA was extracted, and amplicons that spanned the guide binding site were generated by PCR using various Surveyor primer pairs (Table S3). The amplicons were purified using the QIAQuick PCR purification kit (Qiagen). Heteroduplexes were formed by subjecting the purified amplicons to heat treatment followed by gradual cooling to 4 °C. The normalised solutions were treated with Surveyor nuclease according to the manufacturer's instructions. Bands were resolved using 2% agarose gels. The relative band intensity (volume intensity) was quantified using Image Lab software (Bio-Rad) after subtracting the background and adjusting the band limits to the band edges. The percentage of indel mutation induced by Cas9 was calculated as indicated in the study by Ran *et al.* (2013b) using the following formula:

$$\text{Indel}\;\left(\%\right)=100\;\times \;\left[1-{\left(1-f_{\text{cut}}\right)}^{1/2}\right],$$

where $\text{fcut}=\frac{\mathrm{Sum of band intensity of digested products}}{\left(\mathrm{Sum of band intensity of digested}+\mathrm{undigested products}\right)}.$

Gene activation was carried with vectors that simultaneously expressed the sgRNA and an inactive nuclease (dCas9) fused to a tandem repeat of the transactivation domain (TD) of the virion protein (*herpes simplex* virus) VP16 (four in VP64 and 10 in VP160) (Cheng *et al.*, 2013) and/or a vector expressing a hybrid activation domains (dCas9-VPR: VP64, p65, and Rta) (Chavez *et al.*, 2015). Cloning of the sgRNAs (Table S2) into the vectors was performed as described in the previous section. Plasmids used for transient transfections of cells included pAC152-dual-dCas9VP64-sgExpression (sgRNA-dCas9VP64, Addgene plasmid #48238), pAC154-dual-dCas9VP160-sgExpression (sgRNA-dCas9VP160, Addgene plasmid #48240), a gift from Rudolf Jaenisch (Cheng *et al.*, 2013), and Sp-dCas9-VPR (Addgene plasmid # 63798), a gift from George Church.

### Donor vector construction

To construct the donor vector for targeted correction, a 2619 bp insert carrying a PGKpuroΔtk double selection cassette flanked by the *inverted terminal repeat* sequences (ITRs) was excised from the pMCS-AAT-*PB:*PGKpuroΔtk plasmid (kindly provided by Dr. Allan Bradley from Wellcome Trust Sanger Institute, Cambridge, UK) (Yusa *et al.*, 2011a) using *NotI* and *NheI* restriction enzymes. The excised fragment was cloned into a plasmid (pHR/ITR), synthesised by GenScript (http://www.genscript.com), digested with the same enzymes. The plasmid pHR/ITR carries the 5′ and 3′ homology arms (approximately 1 kb for each arm) and a segment of PB inverted terminal repeats to complement the fragment isolated from the pMCS-AAT-*PB:*PGKpuroΔtk plasmid. Three base substitutions and two SNPs, as well as the substitution of the disease-causing mutation, were introduced in the left arm of the donor template sequence [Fig. 1(c)] in otherwise isogenic sequences to the parental beta-thalassemia-iPSCs. Primers used to sequence the *HBB* locus are shown in Table S3.

### Transfections

To induce DNA breaks in HEK293FT, the cells were cultured as previously described and seeded into a 12-well plate (about 130 000/well) one day prior to transfection. 1 *μ*g of the dual sgRNA-Cas9-expressing vector or 300 ng of each of the pair of nickase plasmids was transiently transfected into 293FT cells using Lipofectamine LTX (Invitrogen) according to the manufacturer's instructions. The medium was replaced after 24 h. Cells transfected with the puromycin expressing vector were subjected to drug selection (for two days) starting at 24 h post-transfection. Assessment of guide RNA on-target modifications was carried on cells collected by trypsinisation 72 h post-transfection.

To induce gene activation, HEK 293FT cells were used to screen for the sgRNA vector combination that displayed most robust activation of the *HBB* gene. Cells were transfected as described above with 1 *μ*g of the total sgRNA-dCas9–(VP16)n or 0.1*μ*g of sgRNA-dCas9–(VP16)n and 0.5*μ*g of dCas9-VPR. The amount of sgRNA-dCas9–(VP16)n was equally divided between multiple vectors targeting different sites in the proximal promoter region of the *HBB* gene. Gene expression analysis was carried 72 h post-transfection.

Nucleofection of iPSCs was carried out as described in Yang *et al.* (2013). Briefly, before nucleofection, cells were treated with a 10 *μ*M Y-27632 ROCK inhibitor for at least 2 h. Unless stated otherwise, 2 × 10^6^ iPSCs were collected by trypsinisation and resuspended in 20 *μ*l of Nucleofector mix that included P3 and supplement solutions (4D Nucleofector, Lonza, http://www.lonza.com). The mix was transferred to one well of a nucleocuvette strip that contained an equivalent of 2 *μ*g of the sgRNA-Cas9WT expression plasmid or an equal mixture of plasmids for paired nickase (in a maximum volume of 2 *μ*l) or 1–2 *μ*g of sgRNA-dCas9–(VP16)n in the presence or absence of the 100 ng dCas9-VPR expressing vector. The cells were nucleofected using the CB150 program. For HDR, 500 ng of the donor vector was added to the suspension mixture. Nucleofected cells were plated in one well of a 6-well plate on feeders in KSR-hESC medium supplemented with 10 *μ*M Y-27632 ROCK. The ROCK inhibitor was removed from the medium after 24 h. Puromycin selection to isolate targeted clones was started at 72 h post-transfection, using 1 *μ*g/ml for three days, and then maintained at 0.5 *μ*g/ml until transfection with transposase.

To induce *HBB* activation in K562 cells, 1 × 10^6^ cells were nucleofected with 1.5 *μ*g of the same vector combination used in iPSCs (sgRNA3–8-dCas9-VP64/VPR) using a 4D-Nucleofector X Kit for CD34^+^ cells, Program FF-120 (Lonza) according to the manufacturer's protocol. Cells were collected 24 and 72 h post-transfection.

Excision of the selection cassette was carried by transfecting targeted iPSCs with 2 *μ*g of a plasmid expressing the hemagglutinin-tagged (HA-tag) hyperactive *piggyBac* transposase (kindly provided by Dr. Allan Bradley) using the above-mentioned transfection conditions in iPSCs (Yusa *et al.*, 2011b). FIAU selection was ineffective, as demonstrated by the high survival of a negative control (biallelic targeted clones). A stepwise enrichment approach was adopted which involved two stages [Fig. S4(b)]: the first stage comprised enrichment for excision events using the following: (1) cell pool fractionation, FIAU selection to eliminate cells expressing Δtk (inefficient), and multiplex-PCR-based screening; (2) followed by clonal enrichment by plating the selected cell pool at low density and clones that displayed a prominent signal for the restored allele by multiplex screening [Fig. S4(c)] were selected for further analysis [Fig. S4(d)]. The second stage involved cloning by limiting dilution of cells derived from the selected clones with the highest proportion of positive events [Fig. S4(e)]. The multiplex PCR screening strategy entailed using three different primers [Fig. S4(a)] as described in the study by Yusa (2013): (1) a common gene-specific primer downstream of the insertion site, (2) insertion-specific primers (3′ITR region of *piggyBac*), and (3) a reverse gene-specific primer (Table S3). This strategy allowed the simultaneous amplification of the corrected allele with a restored TTAA site and the presence of the selection cassette.

The absence of reintegration was confirmed in the selected clones using a PCR-based method with a primer pair located inside the puromycin selection cassette (Table S3).

### RT-PCR and qRT-PCR expression analyses

For RNA extraction, cells were lysed in TRIzol reagent (Ambion) followed by purification using the PureLink RNA Mini Kit (Ambion) with on-column DNAase treatment. One microgram of RNA was used for reverse transcription with the iScript cDNA synthesis kit (Bio-Rad, Hercules, CA, http://www.bio-rad.com) according to the manufacturer's specifications. Amplicons for RT-PCR were generated from cDNA samples using PCR conditions specified in the next sections (PCR amplification and sequence verification). Quantitative RT-PCR (q-PCR) analysis was performed in triplicate (or duplicate) using the CFX96 thermal cycler (Bio-Rad) with Ssofast evagreen q-PCR mix (Bio-Rad). More than one housekeeping gene was used to normalise gene expression: *GAPDH* and *ACTB* (β-actin). Expression data were reported as relative gene expression (ΔCt). Sets of primers used to amplify the intended locus are listed in Table S3.

### PCR amplification and sequence verification

Genomic DNA was extracted using the ISOLATE II genomic DNA kit from Bioline according to the manufacturer's instructions. PCR reactions were performed using the following: (1) Phusion high-fidelity DNA polymerase (New England Biolabs) for sequencing of the *HBB* locus; (2) Herculase II fusion polymerase (Agilent Technologies) for sequencing of the targeted clones, HMA, and for the Surveyor assay (Surveyor Mutation Detection Kits); and (3) LongAmp Taq DNA polymerase (New England Biolabs) for the PCR-based screening for the targeted integration of the selection cassette and for screening of the reintegration of the selection cassette. Touchdown PCR was mostly used to amplify the specified locus (25–30 cycles) using cycling parameters and protocols specified by each manufacturer (depending on the Taq polymerase used). Sanger sequencing was performed by the Australian Genome Research Facility (AGRF, Brisbane node). Primer sequences are shown in Table S3.

### PCR-based screening of DNA modifications in iPSC clones

Fifty puromycin-resistant clones were screened for the presence of the targeted allele and/or the original alleles. Two clones from patient iPSCs transfected with sgRNA1/3 and a linear donor vector, 14 clones with sgRNA1/3 and a circular donor, and 34 clones with sgRNA4/5 and a circular donor were included in the analysis (Table I). The screening was carried out as described by Yusa (2013). Briefly, cell lysate was prepared by cutting a small section from clones, suspending it in water with subsequent heat treatment for 10 min. This was followed by incubation for 1 h with proteinase K. Screening for on-target and random integration was performed simultaneously on the same cell lysate using the same PCR conditions described above.

Screening for targeted events was carried out in the presence of two sets of primers (Table S3): one for genotyping the right arm and the other for the left arm [Fig. S2(a)]. Amplification was carried out using LongAmp Taq DNA polymerase as detailed in the Yusa protocol (Yusa, 2013), and the obtained PCR amplicons were analyzed on a 1.5% agarose gel. The band pattern was used to screen for the absence or the presence of a possible targeting event at one or both alleles [Figs. S2(b) and S2(c)]. DNA from the BT-iPSC clone was used as a positive control to screen for the unmodified original allele and also served as a negative control for the targeted allele.

Screening for random integration was performed on the same cell lysate using sets of primers listed in Table S3. The primer pairs were designed as suggested by Yusa's protocol (Yusa, 2013) where each primer set is designed to bind to a DNA region that spans the junction site between the homology arms (left and right arms) and the plasmid backbone [Fig. S2(d)]. Both a negative (beta-thalassaemia iPSCs) and a positive control (donor plasmid) were used to validate the assay. Samples with positive bands at either arm were excluded from further analysis.

Heteroduplex mobility assay (HMA) was used for allelic alteration screening at undesired on- and off-target sites. The screen was carried out in targeted clones as described by Zhu *et al.* (2014), with minor modifications. To screen for undesired on-target mutations, genomic DNA was extracted from both BT-iPSCs and targeted iPSC clones at the same passage number. PCR amplicons for the DNA region flanking the Cas9 targeting sites were amplified from iPSC clones (resistant and BT-clones) and the donor vector using three sets of primers as shown in Fig. S3(a). The first set was used to detect indel in the untargeted allele, and the other two were used to screen the left and the right arms of the targeted allele [Fig. S3(a)]. The primer sequences used are listed in Table S3. PCR products were assessed for the correct size on 2% agarose gel. In order to generate heteroduplexes, PCR products from the control sample (uncorrected BT-iPSCs or donor vector) were mixed with products from the tested sample at a ratio of 1:1 and subjected to denaturing and reannealing procedures. The annealed products were analysed by electrophoresis using 12% native polyacrylamide gels. The gel mixture was prepared using acrylamide-bisacrylamide (29:1, w/w), 1× Tris-borate-EDTA (TBE), ammonium persulfate, and tetramethylethylenediamine (TEMED). Samples were loaded into wells and subjected to a constant voltage of 120 V/cm for ≈2 h. The gel was immersed in diluted SYBER Gold solution for 35 min before visualisation using the Geldoc XR1 Imaging System (Biorad). The allelic alteration was identified by comparing the variability in the dsDNA band pattern to a formed control homoduplex to rule out artefacts produced during PCR amplification. The genotype of selected samples was further validated by Sanger sequencing.

For off-target screens, the same steps as described in the previous section were followed using the primer pairs listed in Table S4. The hybridized products were formed by mixing amplicons obtained from the targeted clone with that obtained from BT-iPSCs.

### Western blot

3.5–4 × 10^6^ K562 cells (untransfected or transfected with dCas9-activators) were washed with ice cold phosphate-buffered saline (PBS) after harvesting. The pellet was suspended in 100 *μ*l Laemmli buffer and heated at 90 °C for 5 min. 20–25 *μ*l of the cell lysate was loaded into wells of SDS-PAGE gel (8%–16% Mini-PROTEAN TGX Stain-Free Protein Gels, Bio-Rad). Electrotransfer to the Polyvinylidene difluoride (PVDF) membrane was carried using the iBlot™ Gel Transfer Device (ThermoFisher scientific). The blot was blocked with TBS-T (Tris buffered saline with Tween) containing 5% non-fat dry milk (Bio-Rad) for 1 h at room temperature, then probed with a primary antibody diluted in the blocking buffer overnight at 4 °C. Subsequently, the membrane was washed and incubated with a secondary, horseradish peroxidase-labeled antibody (Cell Signalling, 1:5000) for 1 h at room temperature, washed, and developed using Clarity ECL (substrate Bio-Rad) according to the manufacturer's instructions. The blots were visualised with the Gel Doc XR+ System (Bio-Rad) and analysed using Image Lab (Bio-Rad) software. Molecular weights were determined by using the Precision Plus Protein Dual Color Standard (Bio-Rad). Packed RBCs lysed in RIPA buffer were used as a positive control for beta-globin. Antibodies used were (1) anti-beta-globin antibody [Santa Cruz Biotechnology (sc-21757, dilution 1:500)], anti-GAPDH antibody (Cell Signalling, 1:3000), Anti-mouse IgG, HRP-linked Antibody (Cell Signalling #7076, 1:5000), and Anti-rabbit IgG, HRP-linked Antibody (Cell Signalling #7074, 1:5000).

## SUPPLEMENTARY MATERIALS

See supplementary materials for HBB locus targeting in HEK293FT (Fig. S1), PCR-based screening puromycin-resistant clones (Fig. S2), screening of on- and off-target mutagenesis in targeted clones (Fig. S3), a description of enrichment and isolation of iPSC clones with a restored TTAA site after excision of the cassette (Fig. S4), Activation of the HBB gene using CRISPRa (Fig. S5), clone numbers and experimental design (Table S1), gRNA sequences used (Table S2), and lists of primers used (Tables S3 and S4).
